# Overlapping clinical features of transfusion-related acute lung injury and anaphylactic shock during general anesthesia: a case report

**DOI:** 10.1186/s40981-026-00867-3

**Published:** 2026-07-04

**Authors:** Masashi Inoue, Akiko Taniguchi, Masato Morita

**Affiliations:** Department of Anesthesiology, Anjo Kosei Hospital, 28-Higashihirokute, Anjo-cho, Anjo-city, Aichi 446-8602 Japan

**Keywords:** Transfusion-related acute lung injury, Anaphylactic shock, Blood transfusion

## Abstract

**Background:**

Transfusion-related acute lung injury (TRALI) and anaphylactic shock are rare but life-threatening transfusion-related complications. Differentiating between these conditions during general anesthesia is difficult because subjective symptoms cannot be assessed and intraoperative diagnostic evaluation is limited.

**Case presentation:**

A 40-year-old woman undergoing surgery for ovarian cancer under general anesthesia developed acute hypoxemia during transfusion. After completion of the transfusion, she developed sudden hypotension, tachycardia, and generalized erythema, which responded promptly to adrenaline administration. Respiratory failure persisted, and frothy sputum appeared in the endotracheal tube. Postoperative chest radiography showed bilateral pulmonary infiltrates, while echocardiography revealed preserved cardiac function. Anti-human leukocyte antigen (HLA) class I and II antibodies were detected in the transfused fresh frozen plasma, and serum tryptase levels were elevated postoperatively.

**Conclusions:**

This case demonstrated overlapping clinical features of TRALI and anaphylactic shock during general anesthesia. Although donor anti-HLA antibodies supported the possibility of TRALI, severe anaphylaxis alone could not be completely excluded as an explanation for the pulmonary edema.

## Background

Transfusion-related adverse reactions remain a significant cause of morbidity and mortality in the perioperative period. Among them, transfusion-related acute lung injury (TRALI) and anaphylactic shock are particularly serious complications that require distinct management strategies [1, 2]. During general anesthesia, the diagnosis of these conditions is difficult because patients cannot report subjective symptoms such as pruritus, throat discomfort, or dyspnea, and intraoperative assessment and diagnostic testing are limited. In addition, hemodynamic and respiratory conditions may be influenced by multiple perioperative factors, further complicating differential diagnosis [3]. Although TRALI and anaphylactic shock are individually uncommon, their concomitant occurrence during transfusion has not been well documented. We present a case with overlapping clinical features of TRALI and anaphylactic shock occurring during general anesthesia, in which differentiation between these conditions was particularly challenging.

## Case presentation

A 40-year-old woman (height: 148 cm, weight: 48 kg) was scheduled to undergo interval debulking surgery and low anterior resection following neoadjuvant chemotherapy for progressive ovarian cancer. She had no underlying diseases, including cardiovascular or respiratory disorders. Her New York Heart Association (NYHA) functional classification was class I, and her Hugh–Jones classification was grade I. No abnormal findings were observed on the preoperative electrocardiogram or chest radiograph. She had no history of allergies and had never received a blood transfusion. Anesthesia was administered as combined epidural and general anesthesia. General anesthesia was induced and maintained with propofol, remifentanil, and rocuronium.

Approximately 3.5 h after the start of surgery, increased surgical bleeding was observed, and laboratory tests revealed a hemoglobin level of 8.1 g/dL and a fibrinogen level of 190 mg/dL. Transfusion was initiated with 300 mL of autologous blood, four units of red blood cells (RBCs), and eight units of fresh frozen plasma (FFP). No abnormalities were observed during transfusion of autologous blood and RBCs. However, five minutes after initiating the second pack of a four-unit FFP preparation, the patient’s percutaneous oxygen saturation (SpO_2_) decreased from 100% to 87% under an inspired oxygen fraction of 0.33. Decreased breath sounds were auscultated over the right lung, and no sputum could be aspirated. Although lung recruitment maneuvers were performed and positive end expiratory pressure (PEEP) was increased from 5 to 10 cmH_2_O, SpO_2_ showed minimal improvement.

**Fig. 1:**
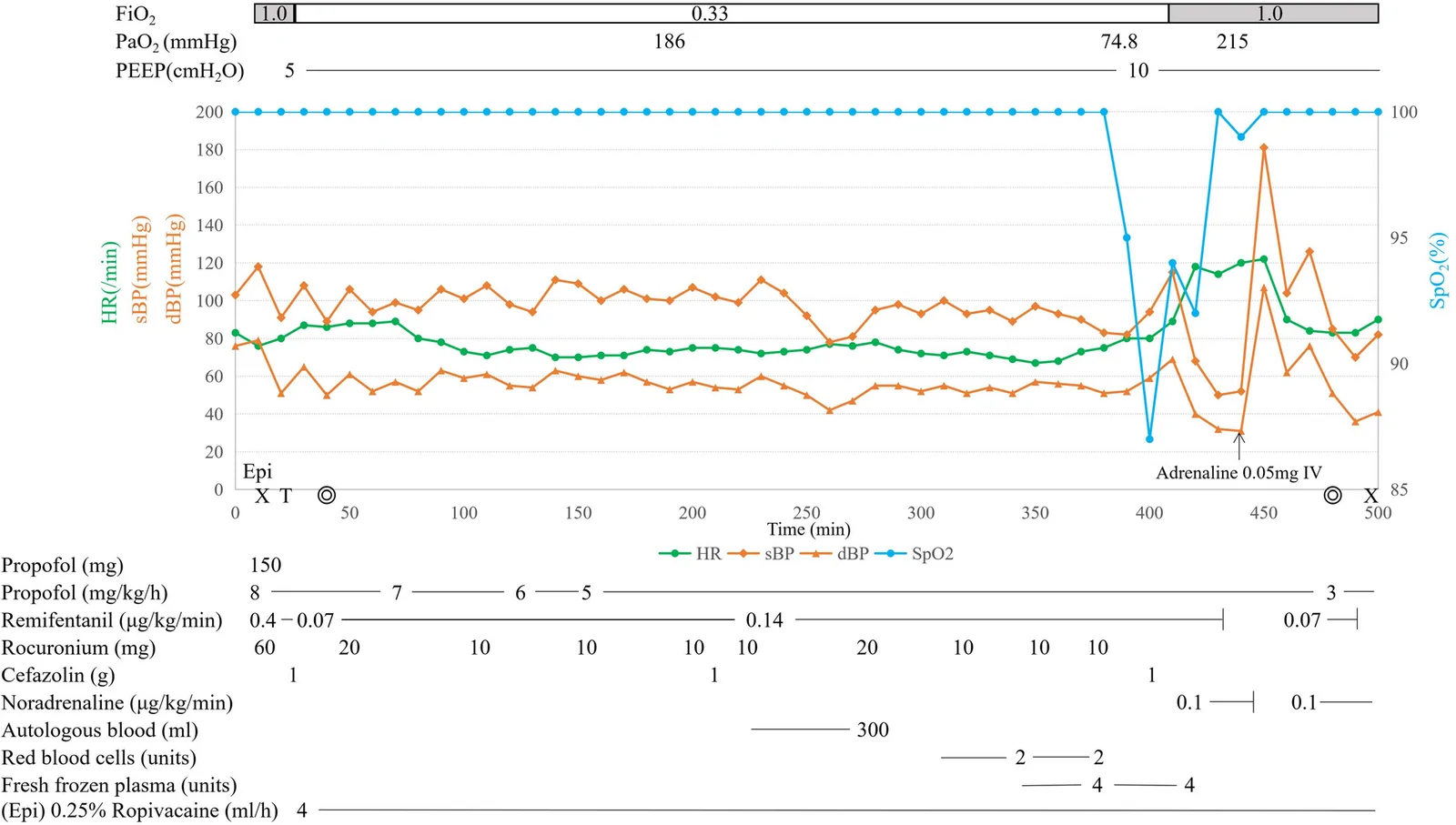
Anesthesia record. A rapid deterioration in respiratory and hemodynamic status was observed after approximately 380 min on the chart. HR, heart rate; sBP, systolic blood pressure; dBP, diastolic blood pressure; SpO₂, percutaneous oxygen saturation; FiO2, inspired oxygen fraction; PEEP, positive end expiratory pressure; X, start or end of anesthesia; T, intubation; Epi, epidural anesthesia; ◎, start or end of surgery

Ten minutes after the blood transfusion was completed, the patient developed sudden hypotension, with systolic blood pressure dropping to the 50 mmHg range, accompanied by tachycardia exceeding 120 bpm. Despite administration of 100% oxygen, fluid resuscitation, ephedrine, phenylephrine and noradrenaline, her blood pressure did not increase. Because erythema was observed from the face to the abdomen, anaphylactic shock was suspected, and 0.3 mg of adrenaline was administered intramuscularly. As the response was insufficient, 0.05 mg of intravenous adrenaline was subsequently administered, which promptly restored blood pressure. Despite hemodynamic stabilization, severe hypoxemia persisted, and a large volume of frothy sputum was noted in the endotracheal tube. Although suctioning sputum resulted in some improvement in respiratory failure, ventilatory management was continued with PEEP at 10 cmH₂O throughout the remainder of the surgery. Despite the patient’s poor respiratory status, the planned surgical procedure was completed as scheduled. The anesthesia chart shows the relationship between hemodynamic changes, respiratory conditions, drug administration, and blood transfusion during general anesthesia (Fig. 1).

**Fig. 2:**
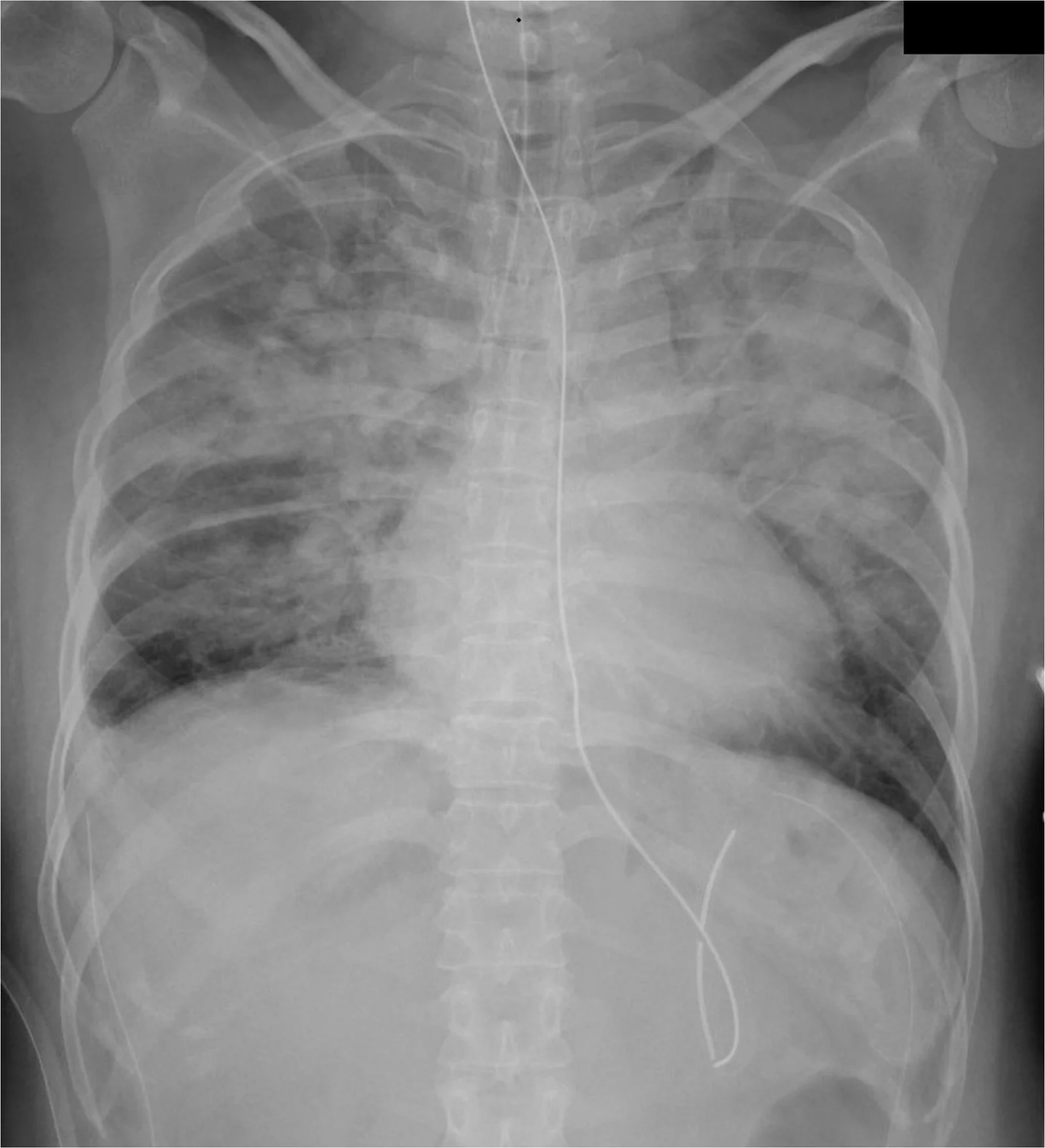
Chest radiograph obtained immediately after surgery. Postoperative chest radiograph revealed bilateral infiltrates in both lung fields, suggestive of pulmonary edema

Postoperative chest radiography revealed marked bilateral pulmonary infiltrates without evidence of cardiac dilation (Fig. 2). The patient was transferred to the intensive care unit (ICU). Echocardiography revealed a small left ventricular cavity with preserved systolic function. In addition, there were no findings suggestive of elevated left atrial pressure, such as left atrial enlargement. The inferior vena cava was not dilated, and respiratory variation was preserved. Furthermore, the patient had no risk factors for acute respiratory distress syndrome (ARDS), such as pneumonia, sepsis, or pancreatitis. TRALI was considered because the patient fulfilled the clinical criteria for possible TRALI type I. However, severe anaphylaxis-associated pulmonary edema could not be completely excluded. Wheals observed on the extremities and trunk supported a diagnosis of anaphylaxis.

**Fig. 3:**
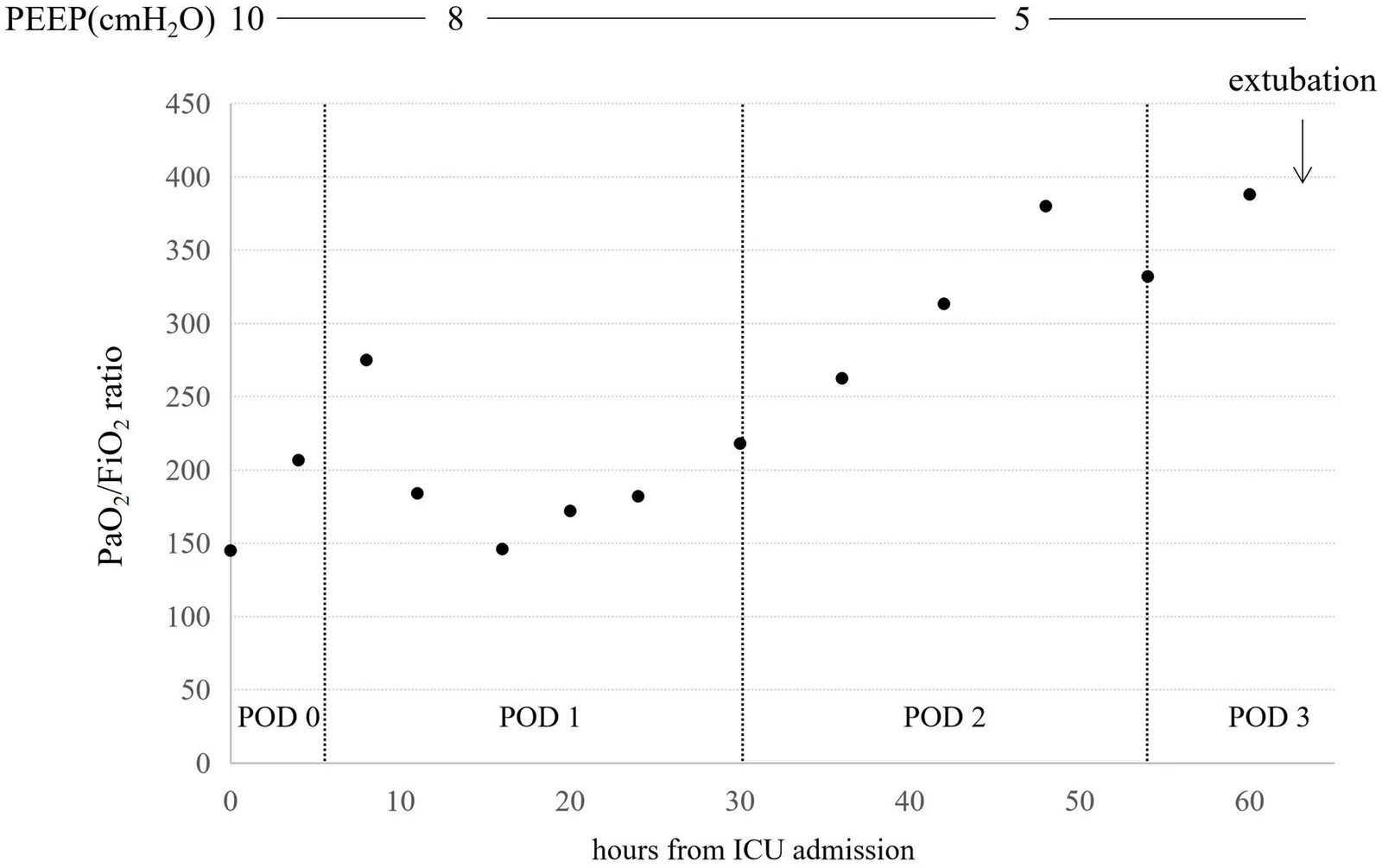
Respiratory status in ICU. The patient’s respiratory status gradually improved over several days in the ICU. FiO_2_, inspired oxygen fraction; PEEP, positive end expiratory pressure; POD, postoperative day

**Fig. 4:**
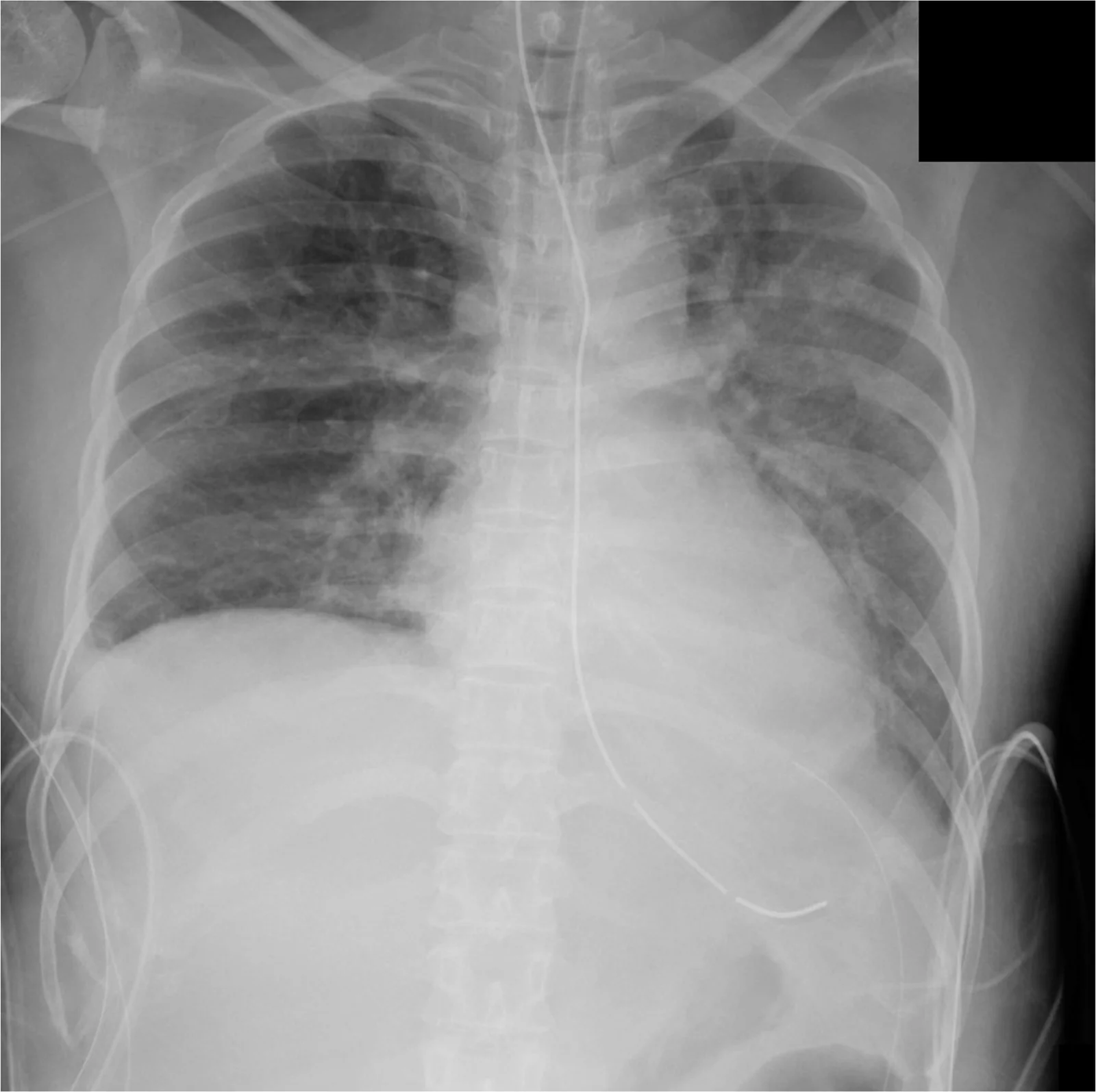
Chest radiograph on postoperative day 3. Chest radiograph on postoperative day 3 demonstrated improvement of the pulmonary infiltrates, suggesting resolution of pulmonary edema

The patient’s respiratory condition began to improve on postoperative day (POD) 2. Fig. 3 shows the course of the patient’s respiratory status until extubation. She was extubated on POD 3 (Fig. 4), discharged from the ICU on POD 4, and discharged from the hospital on POD 20. Postoperative laboratory testing revealed a marked elevation in serum tryptase (20.6 µg/L) compared with the preoperative level (3.3 µg/L), whereas N-terminal fragment of the pro brain natriuretic peptide (NT-pro-BNP) levels remained unchanged (preoperative, 45 pg/mL; postoperative, 22 pg/mL). The patient underwent blood testing for other laboratory parameters on the day before surgery, and the separated plasma sample was stored refrigerated for 1 week according to our institutional policy. This sample was therefore used as the preoperative specimen. The postoperative specimen was collected in the ICU approximately 1.5 h after the onset of suspected anaphylactic shock during surgery. Furthermore, donor-derived anti–human leukocyte antigen (HLA) class I and class II antibodies were identified in the initial four units of fresh frozen plasma administered intraoperatively, using a Luminex-based single-antigen bead assay. The donor of this preparation was female; however, her pregnancy history was unknown. No anti-haptoglobin antibodies, anti-immunoglobulin (Ig) A antibodies, or deficiencies in plasma proteins, including haptoglobin or IgA, were identified in the patient’s preoperative blood samples. These antibody titers were measured using the plasma sample collected on the day before surgery.

## Discussion

We report a case with overlapping clinical features of TRALI and anaphylactic shock during general anesthesia. Both conditions are potentially life-threatening transfusion-related complications. However, they require fundamentally different management strategies, making accurate and timely diagnosis clinically crucial.

TRALI is defined as noncardiogenic pulmonary edema occurring within 6 h of transfusion [1]. Reported risk factors for the development of TRALI include the use of FFP or platelet concentrates, as well as transfused blood products containing anti-HLA antibodies. The use of FFP from female donors, particularly those with a history of pregnancy, has been reported as an important risk factor [1, 4].

Anaphylaxis is a systemic, immediate-type hypersensitivity reaction that requires prompt treatment. Diagnosis during general anesthesia has been suggested to be potentially difficult, because it must rely solely on objective indicators [3]. Therefore, a scoring system incorporating objective parameters for the diagnosis of anaphylaxis has been reported and may be useful in such cases [5]. Moreover, an increase in serum tryptase levels between the preoperative and postoperative periods has been reported to be useful as an adjunctive diagnostic marker [6]. Adrenaline administration should be considered at the time anaphylaxis is suspected.

In this case, hypoxemia, reduced tidal volume, and decreased unilateral breath sounds developed during transfusion, initially leading us to consider atelectasis. Previous studies have demonstrated that atelectasis was consistently identified as the most common and clinically significant cause of hypoxemia during general anesthesia [7, 8]. However, retrospective evaluation suggests that respiratory failure due to TRALI may have already been present at that point. At the time frothy sputum was observed in the endotracheal tube, TRALI or transfusion-associated circulatory overload (TACO) was considered more likely than atelectasis or bronchospasm. In retrospect, performing intraoperative ultrasonography might have been beneficial. Lung ultrasound has been shown to be useful for the diagnosis of pulmonary edema and acute respiratory failure even during general anesthesia, and, when combined with echocardiography, it is also useful for differentiating TACO from TRALI [9]. In particular, transesophageal echocardiography may have been useful in this case because it allows detailed assessment of cardiac function without interference from the surgical field. In TACO, reduced cardiac contractility and dilation of the inferior vena cava may be observed, whereas in anaphylactic shock and TRALI, echocardiography may demonstrate preserved or hyperdynamic left ventricular systolic function due to peripheral vasodilation, although transient myocardial dysfunction has also been reported [10]. Postoperative chest radiography demonstrated bilateral pulmonary infiltrates, and transthoracic echocardiography revealed preserved cardiac function without evidence of volume overload. As described above, these echocardiographic findings were more suggestive of TRALI than TACO. In addition, serum NT-pro-BNP levels were retrospectively compared between the preoperative and postoperative periods to differentiate TRALI from TACO, and no increase was observed, making TACO unlikely. The detection of anti–HLA class I and class II antibodies in the transfused blood products from a female donor further supported the diagnosis of TRALI, and the subsequent clinical course of respiratory failure management in the ICU was consistent with this diagnosis. In summary, the present case involved a patient without risk factors for ARDS who developed sudden hypoxemia during surgery and demonstrated bilateral pulmonary infiltrates without cardiomegaly on postoperative chest radiography. However, echocardiography revealed no clear evidence of fluid overload or cardiac dysfunction, and anti-HLA antibodies were detected in the transfused blood products. Therefore, the patient fulfilled the diagnostic criteria for possible TRALI type I. Nevertheless, the diagnosis of TRALI remains uncertain. Severe anaphylaxis itself may cause non-cardiogenic pulmonary edema through increased vascular permeability and inflammatory mediator-induced pulmonary capillary leak. Therefore, the pulmonary edema observed in this patient could potentially be explained by anaphylaxis alone. Although donor anti-HLA antibodies supported the possibility of TRALI, donor antibody positivity alone is insufficient to establish a definitive diagnosis. Consequently, it remains difficult to determine whether this case represented two distinct transfusion reactions or a single severe transfusion-related reaction with overlapping manifestations.

After completion of transfusion, the patient developed sudden hypotension and tachycardia accompanied by generalized erythema. These clinical findings, together with a marked postoperative elevation in serum tryptase levels compared with preoperative values, strongly suggested an intraoperative allergic reaction. Excluding the continuously administered agents, rocuronium and cefazolin were administered during surgery and were considered potential causes of anaphylaxis with delayed clinical manifestation. Among these agents, rocuronium had been administered repeatedly throughout the procedure before the onset of anaphylaxis, making it a less likely causative agent. In contrast, cefazolin had been administered more closely temporally associated with the onset of anaphylaxis; therefore, we investigated only cefazolin as a potential cause. A skin prick test for cefazolin was performed on POD 15, and the result was negative. However, current literature suggests that skin testing after perioperative drug reactions should ideally be performed at least 4 weeks after the reaction, and many reviews describe 4–6 weeks as the preferred interval to avoid false-negative results associated with the post-anaphylactic refractory period [11–13]. Therefore, the negative result in the present case does not definitively exclude cefazolin as a causative agent. Nevertheless, based on the temporal relationship between transfusion and symptom onset, transfusion-related anaphylaxis remained the most likely diagnosis. The prompt hemodynamic response to adrenaline administration further supported the diagnosis of anaphylactic shock. Furthermore, according to the aforementioned scoring system, the present case scored 9 points for “Cardiovascular,” 0 points for “Respiratory,” 3 points for “Dermal/mucosal,” 5 points for “Combinations,” 3 points for “Timings,” and 12 points for changes in tryptase levels, resulting in a total score of 32 points. This exceeded the threshold score of 21 points for the diagnosis of “Almost certain to be an immediate hypersensitivity reaction.” In this case, the initial dose of adrenaline was administered intramuscularly, although certain perioperative guidelines recommend intravenous administration as the initial route of treatment [3, 14]. As intravenous administration was ultimately required, initiating treatment intravenously might have been preferable.

Cases showing overlapping clinical features of TRALI and anaphylactic shock are rarely reported. This case highlights the importance of considering multiple coexisting transfusion-related complications when acute hypoxemia and hemodynamic instability are observed during or after transfusion under general anesthesia. Although the diagnosis of these conditions under general anesthesia may be difficult, clinicians should closely observe patient vital signs and physical findings and institute appropriate treatment.

In summary, we report a transfusion-related adverse event characterized by overlapping clinical features of TRALI and anaphylactic shock during general anesthesia. Although donor anti-HLA antibodies supported the possibility of TRALI, severe anaphylaxis-associated pulmonary edema could not be completely excluded. This case highlights the diagnostic challenges encountered when acute respiratory and hemodynamic deterioration occurs during transfusion under general anesthesia. Prompt recognition and timely treatment are essential to optimize patient outcomes.

## Data Availability

Data sharing is not applicable to this article as no datasets were generated or analyzed during the current study.

## Abbreviations

ARDS: Acute respiratory distress syndrome
FFP: Fresh frozen plasma
HLA: Human leukocyte antigen
ICU: Intensive care unit
Ig: Immunoglobulin
NT-pro-BNP: N-terminal fragment of the pro brain natriuretic peptide
NYHA: New York Heart Association
PEEP: Positive end expiratory pressure
POD: Postoperative day
RBCs: Red blood cells
SpO_2_: Saturation of percutaneous oxygen
TACO: Transfusion-associated circulatory overload
TRALI: Transfusion-related acute lung injury
